# The relationship of diverse leisure activities with flourishing

**DOI:** 10.3389/fpsyg.2023.1130906

**Published:** 2023-04-17

**Authors:** Steven E. Mock, Bryan Smale

**Affiliations:** Department of Recreation and Leisure Studies, University of Waterloo, Waterloo, ON, Canada

**Keywords:** leisure, wellbeing, flourishing, eudaimonic, subjective

## Abstract

The relationship between leisure and wellbeing is of great interest in the field of leisure studies. Keyes (2002) developed a typology of *flourishing* vs. *languishing* that encompasses subjective, psychological, and social wellbeing and is linked with physical health and functioning. However, little research has been done to show how participation in various forms of leisure might be associated with this flourishing typology. Drawing on data from community data with over 5,000 adult participants, we assessed how leisure is associated with a flourishing typology. For the present analyses, we focus on scales that assessed social leisure (e.g., socializing with friends), cultural leisure (e.g., festival attendance), home-based leisure (e.g., reading books for pleasure), physically active leisure (e.g., moderate or vigorous), and media-based leisure (e.g., time spent playing computer games or watching TV). A flourishing typology was constructed from single-item ratings on life satisfaction (subjective wellbeing), psychological well-being (self-perceptions that one’s life activities are worthwhile), and social wellbeing (sense of belonging). Flourishing was linked to greater participation in cultural, social, home-based, and physically active leisure. Greater time spent playing computer games and watching TV was associated with languishing. Thus, certain forms of leisure reflect flourishing and others are linked with languishing. The nature of these associations remains to be explored, in particular, whether leisure contributes to flourishing or if flourishing facilitates certain forms of leisure participation.

## Introduction

The relationship between leisure and wellbeing is of great interest in the field of leisure studies (Iwasaki, 2007; Mannell, 2007; Mock et al., 2016). From a psychological perspective, the concept of wellbeing has developed over the last few decades (Ryff, 1989; Deci and Ryan, 2008), shaped largely by the positive psychology movement (Seligman and Csikszentmihalyi, 2000). This movement developed as a response to the previously dominant focus in psychology on mental illness and the notion that wellbeing consisted primarily of the absence of mental illness (e.g., Jahoda, 1958; Ryff, 1989). In contrast, positive psychology sees wellbeing in terms of optimal functioning and even flourishing in life (Keyes, 2002; Deci and Ryan, 2008).

Two of the main categories of wellbeing from a psychological perspective include subjective wellbeing (e.g., positive and negative affect, life-satisfaction; Ryan and Deci, 2001) and psychological wellbeing often referred to as eudaimonic wellbeing (e.g., sense of purpose, mastery, and personal growth; Ryff, 1989; Deci and Ryan, 2008). Building on these two main aspects of wellbeing, Keyes (2002) proposed that beyond subjective and psychological wellbeing, social wellbeing (e.g., social integration, sense of making a contribution to society) is a key aspect of functioning. Keyes further developed a typology of *languishing* and *flourishing,* with moderate mental health as a mid-point, that takes into account subjective, psychological, and social wellbeing. Beyond psychological wellbeing, languishing and flourishing have been linked with physical health, activity limitation, and sick days (i.e., languishing with worse health, flourishing with better health; Keyes, 2002). However, little research has been done to show how participation in various forms of leisure might be associated with this languishing/flourishing typology. Thus, we propose to examine the ways diverse forms of leisure may be linked to languishing, being moderately healthy, and flourishing.

### Subjective and psychological wellbeing

In a foundational paper on subjective wellbeing, Diener (1984) describes subjective wellbeing as both the experience of pleasure (positive affect) and judgements about how good or bad one’s life is (life satisfaction). Framed another way, positive and negative affect can be considered aspects of experienced wellbeing and life satisfaction considered as evaluative well-being. Subjective wellbeing has been taken up in numerous efforts to assess national and global wellbeing (Diener, 2000; Kahneman et al., 2004; Helliwell et al., 2022). Interestingly, in a subjective wellbeing measure designed for such population-based assessment, leisure activities are rated among the most affectively rewarding in daily experience reports (e.g., intimate relations, socializing after work, dining; Kahneman et al., 2004) with paid work and commuting rated very low in terms of affective reward. But is there more to wellbeing than happiness and life satisfaction? Ryff (1989) has argued that eudaimonic concepts add to our understanding of wellbeing. Eudaimonic aspects of well-being are in keeping with historic Western philosophical traditions and are reflected in more modern notions of self-actualization (Ryff, 1989). Eudaimonic wellbeing may also stem from life experiences that involve considerable distress and effort such as Frankl and Lasch (1992) *will to meaning*, the notion that growth occurs in conditions of suffering and self-fulfillment needs as outlined by Maslow (1943). Research comparing various aspects of life qualities with ratings of both subjective and psychological wellbeing show compelling similarities and differences in these two approaches to wellbeing. For example, the more people rate life as easy with basic wants and needs met, the happier they are, but these ratings appear to have no bearing on perceptions of the meaningfulness of life (Baumeister et al., 2013). In the same study, spending more time with loved ones and rating relationships as more important than achievements were linked to greater meaningfulness of life, but nevertheless, both happiness and meaningfulness were positively associated with feeling connected to others. Thus, considering subjective and psychological wellbeing together captures a broader spectrum of the human experience and wellbeing than either does alone.

### Flourishing

One potential critique of conceptualizations of both subjective and psychological wellbeing is the over-emphasis on the individual perspective; that is, both are assessments focused on the one’s own experiences and wellbeing that may miss the broader social context. For example, social integration is a crucial aspect of wellbeing with substantial psychological and physical health consequences (Durkheim, 1963; Cohen and Wills, 1985; Baumeister and Leary, 1995). To address this shortcoming, Keyes (1998) developed a multi-faceted measure of social wellbeing to reflect not only the personal but also the social nature of life. The key components of social wellbeing include a sense of belongingness to a community, a sense of making valued contributions to society, and a belief that one’s social world is comprehensible (Keyes, 1998). Keyes then built on the notion that wellbeing includes both personal and social functioning and proposed that taking all three aspects of wellbeing into consideration (i.e., subjective wellbeing, psychological wellbeing, and social wellbeing) helps to identify a continuum of mental health that ranges from languishing to flourishing (Keyes, 2002).

This concept of flourishing was originally developed to operationalize mental health in a way that answers the call that wellbeing is not simply the absence of mental illness (e.g., Jahoda, 1958), but rather, consists of the presence of positive emotional states, eudaimonic wellbeing, and social connection and contribution (Keyes, 2002). Drawing on population-based data with over three thousand adults, Keyes assessed the implications of this model of mental health for mental illness (i.e., depression) and functioning (i.e., activity limitation and sick days; Keyes, 2002). This mental health model consisted of two scales of subjective wellbeing (affect and life satisfaction), six scales of eudaimonic wellbeing (Ryff, 1989), and five scales of social wellbeing (i.e., social acceptance, social actualization, social contribution, social coherence, and social integration; Keyes 1998). Responses to items on each scale were averaged then standardized and tertiles were constructed for each scale. Participants with scores in the lower tertile for one of the two subjective wellbeing scales and six of the eleven eudaimonic and social wellbeing scales were classified as languishing (approximately 17% of the sample). Conversely, participants with scores in the upper tertile for one of the two subjective wellbeing scales and six of the eleven eudaimonic and social wellbeing scales were classified as flourishing (approximately 18% of the sample). The remainder were classified as moderately healthy (approximately 65% of the sample). This typology of mental health comparing languishing, moderately healthy and flourishing showed striking implications for mental illness and functioning. For example, 14% of participants reported a major depressive episode in the past year, but the representation across categories varied widely from almost 28% of those in the languishing group reporting a major depressive episode, 13% in the moderately healthy group, to less than 5% in the flourishing group with a major depressive episode. Similarly reports of any activity limitation was graded from a high of 64% among the languishing group to 42% among the flourishing group and over two sick days per month in the languishing group to none in the flourishing group. This preliminary research on a mental health typology of languishing to flourishing helped to establish its implications for mental illness and functioning.

Additional work has shown the consequences of this mental health typology for mortality. To be specific, in a ten-year follow-up with the same population-based sample in the research described immediately above, less than 1% of those in the flourishing category passed away, compared to over 5% in the languishing category (Keyes and Simoes, 2012). Further, in analyses that controlled for other factors linked to mortality including age, gender, education, race, cardiovascular disease, and smoking, languishing (vs. flourishing) was a statistically significant predictor of mortality. Thus, this flourishing framework represents not only a more thorough assessment of wellbeing than the well-established subjective and psychological wellbeing measures, but has been shown to have substantial implications for mental and physical health.

### Leisure and wellbeing

Given these links between flourishing and multiple aspects of wellbeing, how does it relate to leisure? Although much is known about the ways in which leisure relates to wellbeing (e.g., Mannell, 2007), less research has been done to study intersections with flourishing. Leisure is related to well-being in multiple ways through both main effect pathways (i.e., wellbeing as an outcome of leisure participation), but also indirectly including the role of leisure as a protective factor when coping with stress (Iwasaki, 2007; Mannell, 2007). In a review paper, Hutchinson and Kleiber (2005) outline numerous ways that casual leisure (i.e., low-effort, easily enjoyable forms of leisure; Stebbins, 1982, 1997) enhances wellbeing. For example, casual leisure activities have been found to have self-protection properties (e.g., buffer against immediately stressful experiences), the social nature of many casual leisure activities helps to enhance a sense of social connection, and casual leisure may provide a context for reflection and personal growth experiences (Hutchinson and Kleiber, 2005). Although much of the research Hutchinson and Kleiber drew on to illustrate links between leisure and various aspects of wellbeing was cross-sectional, experimental and longitudinal research help to show causal associations of leisure participation and wellbeing. In a meta-analysis investigating the impact of leisure participation on subjective wellbeing, both experimental studies (e.g., leisure interventions) and longitudinal research with diverse forms of leisure show positive effects on life satisfaction (Kuykendall et al., 2015).

However, which forms of leisure are most consistently associated with wellbeing? To help answer that, a recent longitudinal study that drew on data from multiple time points over a nine-year period with over 12,000 participants in a European sample showed that physically active leisure, socializing, engaging in cultural activities (e.g., attendance at theatre or musical performance), reading and taking holidays predicted greater happiness and life satisfaction (Kuper et al., 2022). However, the same study showed that not all leisure activities were as beneficial. For example, social media use and watching TV did not predict happiness or life satisfaction and were sometimes negatively related to wellbeing. Further, online gaming was negatively related to happiness and a significant predictor of decreased life satisfaction. In sum, previous research shows that some, though not all, forms of leisure have the potential to enhance wellbeing, although little work has been done to examine the ways leisure may be related to flourishing.

### Summary and research questions

Although much research has investigated the various ways leisure relates to wellbeing, little to no work has been done to examine the ways leisure may be linked to the more comprehensive flourishing/languishing typology. Flourishing reflects both the personal and social aspects of wellbeing (Keyes, 2002) and is associated with multiple aspects of mental health, physical health, functioning, and even mortality (Keyes, 2002; Keyes and Simoes, 2012), making it an important typology of wellbeing to investigate in the context of leisure. Drawing on data drawn from a large community-based survey, we examine the ways multiple forms of leisure are associated with languishing, being moderately healthy and flourishing, with an index constructed from measures of subjective, eudaimonic, and social wellbeing. Recent research on leisure and wellbeing (Kuper et al., 2022) suggests certain forms of leisure (e.g., exercise, socializing, cultural activities) may be more likely to be positively linked to wellbeing than others (e.g., computer gaming, TV watching) and this will be investigated.

## Methods

Data were drawn from a wellbeing-focused survey conducted by the Canadian Index of Wellbeing in 2018 in a regional municipality of Southwestern Ontario, Canada with a total population of just over 500,000 people (Canadian Index of Wellbeing, 2016; Smale and Gao, 2019). Participants were recruited through mailed invitations to 40,000 randomly selected households in the region asking that one household member at least 16 years of age whose birthday fell closest to June 1 complete an online survey. The final sample was comprised of 5,025 participants, which represents a response rate of approximately 13.4% after taking into account undelivered and/or incorrectly addressed invitations. Broadly, survey questions assessed demographic characteristics, participation in diverse forms of leisure, wellbeing, and various wellbeing indicators (e.g., civic engagement, natural environment, educational opportunities).

### Demographic characteristics

*Age* was self-reported in years. Response options for *gender* included male, female, and if the participant preferred, a self-identified gender. Marital status options included single (never married), married, common law, separated, divorced, and widowed, which were recoded into two categories: *married/common law* (1) and all other options (0). Education was assessed with six categories ranging from elementary school completion to graduate degree and was recoded as either having completed *college/university* (1) and all other lower levels (0). *Total household income* per year was rated in ten categories beginning with approximately $10,000 intervals for the first five options then $20,000 intervals for the remaining intervals up to 10 ($150,000 or greater).

### Leisure activities

Six categories were created to capture levels of participation in different forms of leisure activities. Participation in *social leisure activities* was measured by calculating the combined average of monthly frequency of participation in the following activities: socializing with friends, going out to movies, going out to clubs or bars, and going so spectator sports events. Participation in *cultural leisure activities* was measured using the average annual frequency of attendance at or visitation to: musical concerts, art galleries/museums, festivals, attending ballet, or dance performances, and live theatre. Participation in *home-based leisure activities* was measured by calculating the average of weekly frequency of engagement in the following activities: reading for pleasure (e.g., books, newspapers, and/or magazines), playing board or card games, doing puzzles (e.g., crosswords, Sudoku, and jigsaw), and hobbies (e.g., knitting, crafts, and woodworking). A measure of *exercise* was based on the mean monthly frequency of participation in both vigorous exercise and light exercise. Finally, participation in *computer games* was represented by typical hours per day spent playing computer games and *television watching* was represented by typical hours per day spent watching TV.

### Flourishing

The flourishing typology was constructed from three single item measures. Subjective wellbeing was assessed with a 10-point life satisfaction scale modeled on a recommendation of the OECD (2013) (i.e., “*How satisfied are you with your life in general?”* rated from 1 = very dissatisfied to 10 = very satisfied). Psychological wellbeing assessed with meaningfulness or life significance using a 10-point scale also recommended by the OECD (i.e., *“Overall, to what extent do you feel things you do in your life are worthwhile?”* rated from 1 = not at all to 10 = completely). The notion that a worthwhile life is a reflection of psychological or eudaimonic wellbeing is supported by research by King and colleagues (King et al., 2016) that shows significance (i.e., a worthwhile life) is a key component of meaning. Finally, social wellbeing was assessed using a 7-point scale measuring sense of belonging (i.e., *“Sense of belonging to community”* rated from 1 = very weak to 7 = very strong). Each wellbeing measure was then re-coded into tertiles (bottom third, mid third, and top third approximately). Finally, participants with at least two of lowest tertiles for any of the wellbeing measures were assigned to the *languishing* category, participants with at least two mid-tertile scores for any of the wellbeing measures were assigned to the *moderate mental health* category, and participants with at least two top-tertiles for any of the wellbeing measures were assigned to the *flourishing* category. The approach we use is an approximation to Keyes (2002) approach but not exactly the same. Keyes’s measure of flourishing was assessed with several scales and while we use it as a model for our own measure, the typology for the present analyses is drawn from three single item measures.

### Analysis plan

Analyses began with the calculation of descriptive statistics (e.g., means, standard deviations, frequencies) for all study variables. Next, to assess the association of the flourishing/languishing typology with participation in diverse forms of leisure, analyses of variance were conducted to compare means for participation rates for the various leisure activities by flourishing category. Finally, two separate logistic regression analyses were conducted to assess the association of leisure participation first with the languishing category (vs. moderate mental health and flourishing) and then with the flourishing category (vs. moderate mental health and languishing). For both sets of analyses, Model 1 included demographic characteristics as control variables and in Model 2, all leisure participation variables were added to assess their relative contributions.

## Results

The average age for the sample was approximately 51 years, over 66% of participants identified as female, and 67% were married or cohabiting (see Table 1). The vast majority (over 79%) had a college diploma or university degree and the average income level was over 6, which corresponds to between $60,000 and $79,999 annually. In terms of leisure activities, on average, participants took part in the four social activities about twice per month, took part in each cultural activity just less than twice (i.e., 1.6) per year, took part in the home leisure activities about three times per week, and had slightly more than 10 monthly instances of vigorous and light forms of exercise (see Table 1). On average, participants spent almost an hour more per day on television watching comparted to playing computer games (2.56 vs. 1.67 respectively; Table 1). Wellbeing ratings tended to be above the mid-point for each type of wellbeing (Table 1). The flourishing typology created roughly equal thirds for languishing (26%), moderately healthy (35%) and flourishing (38%) (see Table 1).

**Table 1:** Means and percentages of demographics, socioeconomic status, leisure activities, wellbeing, and flourishing typology.

Variables	*M* (%)	SD
Demographics		
Age	51.27	16.07
Gender		–
Female	66.45	
Male	32.63	–
Participant self-identified	0.92	–
Married/Cohabiting	67.47	–
Socio-economic status (SES)		
College/University degree	79.53	–
Household income level^a^	6.25	2.70
Leisure activities		
Social	1.95	2.00
Cultural	1.60	1.78
Home	3.26	3.18
Exercise	10.71	8.20
Computer games	1.67	3.76
Television watching	2.56	2.24
Well-being		
Subjective	7.72	2.13
Psychological	7.77	2.02
Social	4.42	1.65
Flourishing typology		
Languishing	26.29	–
Moderately healthy	35.54	–
Flourishing	38.17	–

a An income level of 6.25 is based on the 10 ranked categories from “under $10,000” (1) to “$150,000 an over” (10), which falls within the category of “$60,000 to $79,999.”

### Analysis of variance results

ANOVA results with each of the leisure participation variables by the flourishing typology were statistically significant (i.e., social activities *F* (2,4,940) = 40.28, *p* < 0.001; cultural activities *F* (2,4,933) = 74.58, *p* < 0.001; home leisure *F* (2,4,938) = 27.07, *p* < 0.001; exercise *F* (2,4,902) = 27.53, *p* < 0.001; computer games *F* (2,4,858) = 3.50, *p* < 0.05; and television watching *F* (2,4,603) = 20.71, *p* < 0.001). Mean participation scores in each category of leisure activity by flourishing typology are shown in Table 2 and post-hoc comparisons are noted with superscripts. The general pattern shows that in the flourishing group, means for social, cultural, home, and exercise forms of leisure tend to be higher than the moderately mentally health and significantly higher that the languishing groups. Conversely, means in the languishing group for playing computer games and watching television are significantly higher than mean ratings for these activities in the flourishing group.

**Table 2:** Comparison of means for leisure activities by languishing, moderately healthy, and flourishing typology.

Leisure Activities	Languishing	Moderately healthy	Flourishing
	*M*/(SD)	*M*/(SD)	*M*/(SD)
Social	1.54*^b^* (1.98)	2.04*^a^* (2.04)	2.16*^a^* (1.94)
Cultural	1.12*^c^* (1.37)	1.65*^b^* (1.77)	1.89*^a^* (1.97)
Home	2.91*^b^* (3.44)	3.09*^b^* (3.01)	3.68*^a^* (3.10)
Exercise	9.30*^b^* (8.10)	10.99*^b^* (8.45)	11.42*^a^* (7.91)
Computer games	1.91*^b^* (4.17)	1.60 (3.66)	1.57*^a^* (3.55)
Television watching	2.91*^b^* (2.78)	2.45 (2.08)	2.41*^a^* (1.93)

Within Leisure Activities, Tukey LSD *a* > *b* > *c* at minimum significance level *p* < 0.05.

### Logistic regression analyses

For the analysis of languishing (compared to moderate mental health and flourishing), the older participants were, being married or cohabiting, and having greater income were all associated with lower likelihood of languishing (see Table 3, Model 1). With the addition of the leisure activities, social, cultural, and exercise forms of leisure were also associated with lower likelihood of languishing. However, greater television watching was associated with a higher likelihood of languishing (see Table 3, Model 2).

**Table 3:** Binary logistic regression models showing the association of demographic characteristics and leisure activity participation with *languishing* (vs. moderate health and flourishing).

	Model 1			Model 2		
	*B*	SE	Wald	*B*	SE	Wald
Constant	1.21***	0.18	47.33	2.06***	0.00	92.11
Age	−0.02***	0.001	73.36	−0.03***	0.09	93.99
Gender/Female	−0.03	0.09	0.10	−0.02	0.40	0.04
Gender/Self-identified	0.14	0.38	0.14	0.19	0.09	0.23
Married/Cohabiting	−0.27**	0.09	8.90	−0.42***	0.10	20.10
Coll/Univ. degree	−0.12	0.10	1.52	0.01	0.08	0.00
Income	−0.17***	0.02	94.93	−0.14***	0.02	62.88
Social	–	–	–	−0.24***	0.03	66.80
Cultural	–	–	–	−0.18***	0.03	30.94
Home	–	–	–	0.01	0.02	0.05
Exercise	–	–	–	−0.02***	0.01	14.72
Computer games	–	–	–	0.02	0.01	2.23
Television Watching	–	–	–	0.06***	0.02	11.36

*p < 0.05, **p < 0.01, ***p < 0.001.

Somewhat the inverse was found for logistic regression analyses with flourishing (compared to moderate mental health and languishing). The older participants were, being female (vs. male), being married, and having a higher level of income were all associated with a greater likelihood of flourishing (see Table 4, Model 1). Again, social leisure, cultural leisure, and exercise were associated with a greater likelihood of flourishing and television watching was associated with a lower likelihood of flourishing (see Table 4, Model 2).

**Table 4:** Binary logistic regression models showing the association of demographic characteristics and leisure activity participation with *flourishing* (vs. moderate health and languishing).

	Model 1			Model 2		
	*B*	SE	Wald	*B*	SE	Wald
Constant	−3.05***	0.00	259.39	−3.38***	0.21	255.04
Age	0.03***	0.07	151.39	0.03***	0.00	150.26
Gender/Female	0.17*	0.43	5.05	0.16*	0.08	4.68
Gender/Self-identified	−0.13	0.08	0.09	−0.18	0.44	0.16
Married/Cohabiting	0.27***	0.09	10.23	0.34***	0.09	15.90
Coll/Univ. degree	0.10	0.01	1.10	0.01	0.10	0.00
Income	0.12***	0.02	55.44	0.10***	0.02	40.18
Social	–	–	–	0.12***	0.02	35.58
Cultural	–	–	–	0.06**	0.02	8.04
Home	–	–	–	0.01	0.01	0.43
Exercise	–	–	–	0.01**	0.00	8.28
Computer games	–	–	–	−0.01	0.01	1.70
Television watching	–	–	–	−0.07***	0.02	11.73

*p < 0.05, **p < 0.01, ***p < 0.001.

## Discussion

We set out to examine the association of diverse forms of leisure with the flourishing model of wellbeing that represents a comprehensive assessment of both personal and social aspects of wellbeing. To be specific, drawing on community-based survey data with extensive measures of leisure participation, we adapted measures of subjective wellbeing, psychological wellbeing, and social wellbeing to develop a typology of languishing, moderate mental health, and flourishing (Keyes, 2002). In both bivariate analyses (i.e., ANOVA) and logistic regression models controlling for selected demographic characteristics, leisure activities were associated with both flourishing and languishing, but in different ways depending on the activity. Greater participation in social activities such as socializing with friends, more frequent engagement in cultural activities like attending musical concerts or visiting museums, and more physical exercise were associated with higher likelihood of flourishing and lower likelihood of languishing. Conversely, the more time spent watching television, the less likely people were to be in the flourishing category and more likely they were to be in the languishing category.

The flourishing typology was developed as an answer to the call for a measure of wellness that reflected more than just the absence of mental illness but the presence of wellbeing and functioning (Jahoda, 1958; Keyes, 2002). Flourishing has been shown to be associated with key aspects of wellbeing, health and functioning (Keyes, 2002), and the present study represents one of the first efforts we are aware of to link diverse forms of leisure to this model of flourishing. It is worth noting that some of the forms of leisure we assessed (e.g., social leisure, Tables 3, 4) were as strongly associated with languishing and flourishing as household income, helping to reinforce the value of leisure for health and wellbeing. We also found similar patterns to recent longitudinal research that found exercise, socializing, cultural activities and exercise were positively linked to wellbeing but computer gaming and television watching were not (Kuper et al., 2022). Finally, the way we operationalized leisure participation also shows that structural approaches to assessing leisure participation have implications for subjective aspects of wellbeing.

Although our assessment of flourishing was not as thorough as Keyes’ measure (Keyes, 2002), the population-based study that was drawn on for Keyes’ study did not have measures of diverse forms of leisure. Further, our findings are consistent with recent research that shows similar associations between diverse forms of leisure and wellbeing (Kuper et al., 2022), but future research is needed to assess potentially causal associations between leisure and flourishing. We hope that the present research represents a starting point for further research on the potential links between leisure and flourishing and its important consequences for wellbeing and functioning.

## Data availability statement

The data analyzed in this study is subject to the following licenses/restrictions: Request access from the Canadian Index of Wellbeing. Requests to access these datasets should be directed to smale@uwaterloo.ca.

## Ethics statement

The studies involving human participants were reviewed and approved by University of Waterloo. Written informed consent to participate in this study was provided by the participants’ legal guardian/next of kin.

## Author contributions

SM contributed to theoretical development of the manuscript and BS provided the data. All authors contributed to the article and approved the submitted version.

## Conflict of interest

The authors declare that the research was conducted in the absence of any commercial or financial relationships that could be construed as a potential conflict of interest.

## Publisher’s note

All claims expressed in this article are solely those of the authors and do not necessarily represent those of their affiliated organizations, or those of the publisher, the editors and the reviewers. Any product that may be evaluated in this article, or claim that may be made by its manufacturer, is not guaranteed or endorsed by the publisher.
